# The Mitochondria-Mediate Apoptosis of Lepidopteran Cells Induced by Azadirachtin

**DOI:** 10.1371/journal.pone.0058499

**Published:** 2013-03-13

**Authors:** Jingfei Huang, Chaojun Lv, Meiying Hu, Guohua Zhong

**Affiliations:** 1 Key Laboratory of Pesticide and Chemical Biology, Ministry of Education, Laboratory of Insect Toxicology, South China Agricultural University, Guangzhou, P.R. China; 2 Institute of Coconut, Chinese Academy of Tropical Agricultural Sciences, Wenchang, Hainan Province, China; University of Dayton, United States of America

## Abstract

Mitochondria have been shown to play an important role in apoptosis using mammalian cell lines. However, this seems not to be the case in *Drosophila*, an insect model organism; thus more in-depth studies of insect cell apoptosis are necessary. In the present study, mitochondrial involvement during azadirachtin- and camptothecin-induced apoptosis in *Spodoptera frugiperda* Sf9 cells (isolated from *Spodoptera frugiperda* pupal ovarian tissue) was investigated. The results showed that both azadirachtin and camptothecin could induce apoptosis in Sf9 cells. Reactive oxygen species (ROS) generation, activation of mitochondrial permeability transition pores (MPTPs) and loss of mitochondrial membrane potential (MMP) were observed very early during apoptosis and were followed subsequently by the release of cytochrome-c from the mitochondria. Furthermore, the results also revealed that the opening of MPTPs and the loss of MMP induced by azadirachtin could be significantly inhibited by the permeability transition pore (PTP) inhibitor cyclosporin A (CsA), which was used to identify the key role of mitochondria in the apoptosis of Sf9 cells. However, in camptothecin-treated Sf9 cells, CsA could not suppress the opening of MPTPs and the loss of MMP when apoptosis was induced. The data from caspase-3 and caspase-9 activity assays and detection of apoptosis by morphological observation and flow cytometry also uncovered the different effect of CsA on the two botanical apoptosis inducers. Although different mechanisms of apoptosis induction exist, our study revealed that mitochondria play a crucial role in insect cell line apoptosis.

## Introduction

Apoptosis is an evolutionarily conserved form of programmed cell death (PCD) that can be induced by particular endogenous and exogenous factors, such as toxins, hormones, growth factors, nitric oxide, cytokines, heat, irradiation, nutrient deprivation, viral infection, hypoxia [1] and increased intracellular calcium concentration [2]. It is also a basic dynamic process, which is essential to eliminate unwanted or abnormal cells and plays an important role in the stability of the internal environment and the development of multicellular organisms [3], [4]. It is well known that mitochondria play an important role in the intrinsic pathway of mammalian apoptosis by releasing death factors, such as cytochrome-c, a soluble protein, into the cytosol after sensing catastrophic cellular changes [5]. Once released from the mitochondria, cytochrome-c binds apoptotic protease activating factor-1 (Apaf-1) and ATP and subsequently binds to pro-caspase-9 to create a protein complex known as an apoptosome. The apoptosome cleaves pro-caspase-9 to its active form of caspase-9, which in turn activates its effector, caspase-3, and irreversibly commits cells to death [6].

Insects are among the most diverse group of animals on the planet, include more than a million described species and represent more than half of all known living organisms [7]. A majority of the studies on the apoptotic regulation of insect development have been carried out using the *Drosophila* system [8]–[12]. However, the mechanism of apoptosis in insects is not yet clear and still requires further investigation. For example, the role of mitochondrial release of cytochrome c is controversial [13]. More investigations of apoptosis in other insect species should be conducted. Furthermore, it is necessary to study the role of apoptosis in pathological conditions as well as in the development of insects. As one of the most representative commercial Lepidopteran cell lines used for expression of recombinant proteins from baculovirus expression systems, Sf9 cell is another good model for the study of apoptosis [14]. More and more studies on Lepidopteran cell apoptosis regulation, which is induced by different stimuli such as viral infection, irradiation and heavy metal ions have carried out using Sf9 cell, because of its sensitivity for stimuli, stability for infinite culture and close evolutionary genetics to *Drosophila* [14], [15]. However, as we know, there are only a few studies on the apoptosis pathway in Lepidopteran or even in other insect species induced by botanical pesticide, also a strong inducer of apoptosis [16], [17]. Camptothecin is a cytotoxic quinoline alkaloid that inhibits the DNA enzyme topoisomerase I (Topo I) and induces early double-strand breaks in replicating cellular DNA. It was discovered in 1966 in a systematic screening of natural products for use as anticancer drugs [18]–[21]. A previous study found that both camptothecin and actinomycin-D can induce apoptosis in the *Spodoptera frugiperda* Sf9 cell line within a short time and at a low concentration [22]. However, there is no confirmation that the mechanism of apoptosis induced by camptothecin in insect cells is the same as that in mammalian cells.

As a natural insecticidal tetranortriterpenoid mainly obtained directly from seeds of the neem tree (*Azadirachta indica* A. Juss), azadirachtin is relatively safe to most mammals [23] and is currently exploited in agriculture for pest control because it causes potent antifeedant effects [24], growth regulation [25], ovipositional defects [26], sterilant effects and chitin and enzyme inhibition in more than 200 insect species [27]–[29].

Although azadirachtin's mode of action in insect cells is still unknown and requires further investigation, some previous studies may provide helpful clues for the investigation. At the earliest, Rembold et al. [30] indicated that azadirachtin could inhibit Sf9 cell proliferation and protein synthesis. Salehzadeh et al. [31] found that azadirachtin had moderate to strong cytotoxicity, with antimitotic effects in Sf9 cells, which is similar to taxol and colchicine, and it is thought to target tubulin. In the *Drosophila* system, Anuradha et al. [32] reported that azadirachtin could alter or prevent the formation of new assemblages of organelles or the cytoskeleton, especially in the eye and wing imaginal discs of *D. melanogaster* third instar larvae, and suggested that the actin cytoskeleton is targeted by azadirachtin in cells of the *Drosophila* compound eye [33]. Notably, a putative azadirachtin-binding hsp60 complex was identified in *Drosophila* Kc167 cells [34].

Interestingly, recent reports also showed that azadirachtin exerts antitumour effects by inducing cell cycle arrest and mitochondria-mediated apoptosis by regulating proteins involved in cell cycle progression and transducing apoptosis by both the intrinsic and extrinsic pathways, such as proliferating cell nuclear antigen (PCNA), p21^waf1^, cyclin D1, glutathione S-transferase pi (GST-P), NF-κB, inhibitor of κB (IκB), p53, Fas, Bcl-2, Bax, Bid, Apaf-1, cytochrome C, survivin, caspase-3, −6, −8 and −9 and poly (ADP-ribose) polymerase (PARP) [35]–[37]. Furthermore, azadirachtin has anti-inflammatory effects through its interaction with the tumour necrosis factor (TNF) binding domain of its receptors thus inhibiting TNF-induced biological responses, which may be beneficial for anti-inflammatory therapy [38]. Therefore, as an old botanical pesticide and a new antitumour drug, azadirachtin is well worth investigating.

In our study, we investigated the apoptosis-inducing effects of two botanical chemicals, azadirachtin and camptothecin, and confirmed the role of mitochondrial dysfunction in the apoptosis pathway in Lepidopteran cells.

## Materials and Methods

### 2.1. Fluorescent probes and chemicals

All fluorescent probes, including calcein-AM, propidium iodide, rhodamine-123 (Rh123) and 5, 6-chloromethyl-2′, 7′-dichlorodihydrofluorescein diacetate (CM-H_2_DCFDA), were obtained from Sigma-Aldrich (St. Louis, MO, USA). Anti-cytochrome-c antibodies with broad species specificity, a caspases-3 activity assay kit (Ac-DEVD- *p*NA) and a caspase-9 (Ac-LEHD- *p*NA) activity assay kit were obtained from BD Pharmingen (San Diego, CA, USA). Cyclosporin A (CsA), azadirachtin (95%) and camptothecin (95%) were also purchased from Sigma-Aldrich (St. Louis, MO, USA).

### 2.2. Cell culture and treatments

Sf9 cells, obtained from the State Key Laboratory for Biocontrol/Institute of Entomology, Sun Yat-Sen University, Guangzhou, China, were maintained as a monolayer in 25-cm^2^ culture flasks at 26°C in Grace's insect cell medium (Gibco, USA) supplemented with 3.33 g l^−1^ lactalbumin hydrolysate (Oxoid, England), 3.33 g l^−1^ yeastolate (Oxoid, England), 3.33 g l^−1^ tryptone (Oxoid, England), 0.35 g l^−1^ NaHCO_3_ and antibiotics (penicillin-sodium salt 200,000 units/l, streptomycin sulphate 100,000 units/l, kanamycin 50,000 µg/l; Sigma, USA). Growth medium (pH 6.2) was prepared by adding 10% heat-inactivated fetal calf serum (FBS) (Gibco, USA) and stored below 4°C. The doubling time under optimum conditions was found to be 18–24 h, and cells were subcultured every 3 d. Cell numbers were determined with a haemocytometer, and cell viability was determined by trypan blue exclusion.

Cells were treated with 0.75 µg/ml azadirachtin and 1 µM camptothecin (0.1 mg/ml azadirachtin and 0.1 mM camptothecin stock solution prepared in growth medium with 0.5% dimethylsulfoxide (DMSO) for different times. For the CsA assay, Sf9 cells were treated with 3 µM CsA for 1 h. Then, the medium was replaced with fresh medium containing 0.75 µg/ml azadirachtin and 1.0 µM camptothecin for different times.

### 2.3. Live cell morphology and apoptosis

Apoptotic morphological characteristics of Sf9 cells were recorded with an inverted phase contrast microscope (IPCM) (Olympus, Japan) at various times after treatment. Cells cultured with 0.5% DMSO were used as the control. Apoptotic DNA degradation was studied by DNA gel electrophoresis. For DNA gel electrophoresis, DNA fragmentation was extracted with a “minibest blood & cultured cells genomic DNA extraction kit” (Takara, Japan). For analysis, DNA electrophoresis was performed on 1.5% (w/v) agarose gels containing 0.5 µg/ml ethidium bromide.

### 2.4 Measurement of intracellular ROS

Intracellular ROS levels were measured using 5, 6-chloromethyl-2′, 7′-dichlorodihydrofluorescein diacetate (CM-H_2_DCFDA) [39]. After treatment, cells were washed twice with PBS and held at 37°C in phosphate-buffered salines (PBS) containing Ca^2+^, Mg^2+^ and H_2_DCFDA (10 µg/ml; Merck, England). After 30 min, cells were washed again and analysed using flow cytometry.

### 2.5 Calcein loading assay

Mitochondrial permeability transition pore (MPTP) opening was measured directly using a combination of calcein AM and CoCl_2_. Calcein AM, which fluoresces upon binding with Ca^2+^, was used to detect transient MPT pore opening in the high conductance mode of intact cells [40]. This was achieved by monitoring changes in mitochondrial Ca^2+^ levels in the presence of CoCl_2_, which quenched the cytosolic Ca^2+^ signal produced by calcein. In general, cultured cells were harvested by trypsinisation and resuspended at a concentration of 1×10^6^ cells/ml in pre-warmed Hank's Balanced Salt Solution (HBSS) containing 400 µM Ca^2+^. The cell suspension was simultaneously loaded with 2 µM calcein-AM and 400 µM CoCl_2_. After incubation at 37°C for 15 min, the cells were washed with HBSS/400 µM Ca^2+^ to remove excess staining and quenching reagents. The cells were resuspended in HBSS/400 µM Ca^2+^ and analysed using flow cytometry as above.

For fluorescence microscopy, cells were grown on coverslips for 2 d before treatment with azadirachtin and camptothecin. At the end of the treatments, coverslips were briefly rinsed in PBS and loaded with 2 µM calcein-AM and 400 µM CoCl_2_. After incubation at 37°C for 15 min, the cells were washed with HBSS/400 µM Ca^2+^ to remove excess staining and quenching reagents. Cells were examined under a fluorescence microscope (Olympus, BX51), and the change in fluorescence intensity was measured with excitation at 488 nm and emission at 525 nm. Data analysis was performed using image express software.

### 2.6 Mitochondrial membrane potential (Δψm) analysis

Variations of mitochondrial membrane potential were assessed using the fluorescent cationic dye Rh123 [5], a cationic membrane-permeant fluorescent probe, which accumulates in mitochondria as a direct function of the membrane potential and is released upon membrane depolarisation [41]. Mitochondrial membrane potential during Sf9 apoptosis was analysed with flow cytometry and a fluorescence microscope. For flow cytometry, treated Sf9 cells were harvested, washed twice with PBS (pH 7.4) and stained with Rh123 (2 µg/ml) for 30 min at 27°C in the dark. Cells were collected by centrifugation after two PBS washes to remove extracellular Rh123. The fluorescence intensity was analysed with flow cytometry (BD. FACSCalibur) at an excitation wavelength of 470–490 nm and an emission wavelength of 515–565 nm. Data analysis was performed using the CellQuest software program. Rh123 staining was also studied with fluorescence microscopy as described in section 2.5 with few modifications.

### 2.7 Preparation of sub-fractions from Sf9 cells

Sf9 (1×10^7^ cells/ml) cells were harvested at various time points after treatment by centrifugation at 800×g for 10 min and washed twice with cold PBS. The cells were suspended in 200 µl modified lysis buffer (200 mM HEPES-KOH, pH 7.5, 10 mM KCl, 1.5 mM MgCl_2_, 1 mM EDTA-Na_2_, 1 mM DTT, 0.1 mM PMSF, 250 mM sucrose), homogenised with a glass homogeniser, and centrifuged at 1000×g to separate the nuclear fraction. The pellet and supernatant were separated. The pellet was resuspended in 150 µl lysis buffer, sonicated and subjected to centrifugation at 12,000×g at 4°C for 1 h. The resulting supernatant was saved as the mitochondrial fraction. The supernatant was collected and centrifuged again for 20 min at 12,000×g. This subsequently generated supernatant was re-centrifuged at 10,000×g for 1 h at 4°C to remove the residue and saved as the cytosolic fraction.

### 2.8 Mitochondrial swelling

Mitochondria (0.4 mg of protein) was added in a standard medium containing 125 mM sucrose, 65 mM KCl, 10 mM Hepes/KOH, pH 7.4, at 30°C plus 5 mM potassium succinate, 2.5 µM rotenone and 10 µMCaCl_2_ (final volume 1.5 ml) in the presence or absence of palladacycles. The mitochondrial swelling was estimated from the decrease in the relative absorbance at 540 nm in a Beckman DU 640 spectrophotometer (Beckman Coulter, Fullerton, CA).

### 2.9 Mitochondrial respiration

Aliquots of mitochondria (1 mg/ml) were used in measurements of respiratory activity using a Clark-type oxygen electrode (Hansatech Oxygraph) as previously described. Oxygen electrode buffer (130 mM KCl, 2 mM KH_2_PO_4_, 3 mM HEPES, 2 mM MgCl_2_, 1 mM EGTA) was incubated for 1 min in a magnetically stirred chamber at 28°C. The respiratory substrates, glutamate (5 mM) and malate (2.5 mM), were added followed by the isolated mitochondria (200 µg). Basal respiration was first measured in the absence of ADP for 1 min. Subsequently, State 3 respiration was measured in the presence of ADP (0.3 M) to determine the maximal rate of coupled ATP synthesis. Then State 4 respiration was induced by addition of the adenine nucleotide translocator inhibitor atractyloside (50 µM). The respiratory control ratio was calculated using the ratio of State 3 to State 4 respiratory rates.

### 2.10 Western blotting

Cytochrome-c release was analysed using western blotting. The proteins obtained following all treatments were quantified by BCA assay (Invitrogen, USA) and boiled in 2× sample buffer (0.6 M Tris–HCl, 10% glycerol, 2% SDS, 5% β-mercaptoethanol) for 5 min for Western blot analysis. Equal amounts of protein were loaded from each fraction, separated on a 15% polyacrylamide gel and transferred to a PVDF membrane. Afterwards, the membrane was blocked with 5% BSA and probed with an anti-cytochrome-c antibody (1∶2500) or anti-caspase-3 antibody (1∶2000) with broad species specificity (BD, Pharmingen). After three washes in TBS containing 0.1% Tween-20 (TBST), the membrane sample was incubated with anti-mouse IgG-HRP for 1 h. Then, the membrane was washed again, and proteins were detected using with 3′, 3′ -diaminobenzidine (DAB, Invitrogen).

### 2.11 Assessments of caspase-9 and caspase-3 activity

Caspase-3 and caspase-9 protease activities were determined using a commercially available kit according to the manufacturer's instructions. In brief, at various times after treatment, 2×10^6^ cells were collected and washed twice with cold PBS (pH 7.4). The cells were resuspended in 50 µl chilled cell lysis buffer; cells were then incubated on ice for 10 min and centrifuged for 1 min in a microcentrifuge (10,000×g). The supernatants were subsequently transferred (cytosolic extract) to a fresh tube and put on ice for immediate assay or aliquoted and stored at −80°C for future use. Next, 50–200 µg diluted protein was added to 50 µl cell lysis buffer for each assay. Then, 50 µl 2× reaction buffer and 5 µl 4 mM DEVD-*p*NA (caspase-3)/LEHD-*p*NA (caspase-9) was added and incubated at 37°C for 1–2 h. Samples were read at 405 nm in a microtiter plate reader.

### 2.12 Statistical analysis

All experiments were performed in four replicates and were repeated at least four times. Representative experiments or mean values ± SEM are shown in the figures. Significant differences were determined by ANOVA followed by Duncan's multiple range test, and a significant difference was taken as *P<0.05 or **P<0.01.

## Results

### 3.1. Azadirachtin and camptothecin induce apoptosis in Sf9 cells characterised by apoptotic body formation and DNA fragmentation

**Figure 1:**
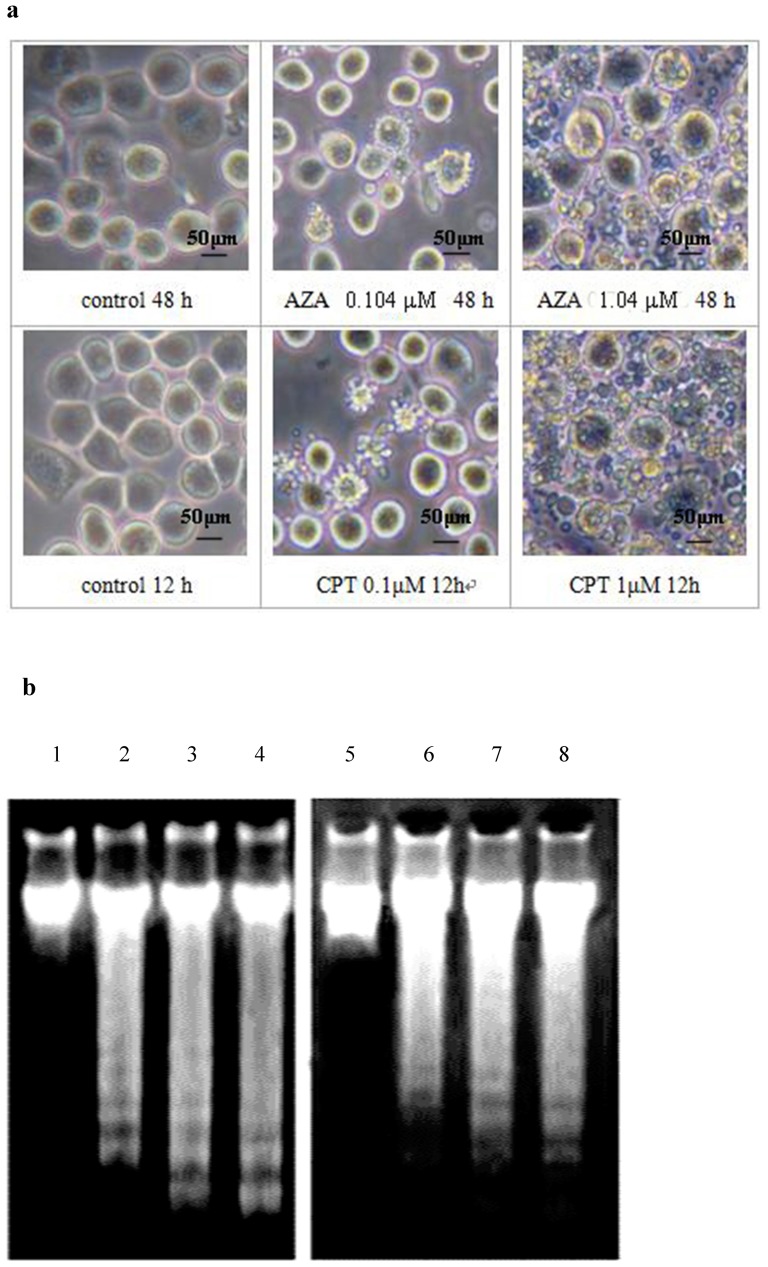
AZA and CPT induced cell apoptosis in Sf9 cells is marked by apoptotic body formation and apoptotic DNA degradation. Apoptotic body formation was observed 48 h and 12 h onwards after treatment with AZA and CPT, respectively. Bar =  50 µm (a). Non-random DNA degradation, which is generally seen during apoptosis, was observed by DNA gel electrophoresis. Lanes 1∼4: control, 0.1 µM, 1 µM and 10 µM of CPT at 12 h after treatment, respectively. Lanes 5∼8: control, 0.104 µM, 1.04 µM, and 10.04 µM of AZA at 48 h after treatment, respectively (b).

The induction of apoptosis by azadirachtin and camptothecin was confirmed by studying apoptotic body formation and apoptotic DNA degradation. And as shown in Fig. 1a, the formation of apoptotic bodies was observed at12 h and 48 h post-treatment in camptothecin- and azadirachtin-treated cells, respectively. Under agarose gel electrophoresis, Fig. 1b also showed non-random, inter-nucleosomal degradation of DNA in azadirachtin- and camptothecin-treated cells. In this study, the apoptotic DNA fragmentation evident from the formation of a typical DNA ladder was observed at 12 h and 48 h post treatment after captothecin- and azadirachtin-treatement, which further confirmed the induction of apoptosis.

### 3.2 Azadirachtin and camptothecin treatment results in an increase in ROS levels and cytochrome-c release

**Figure 2:**
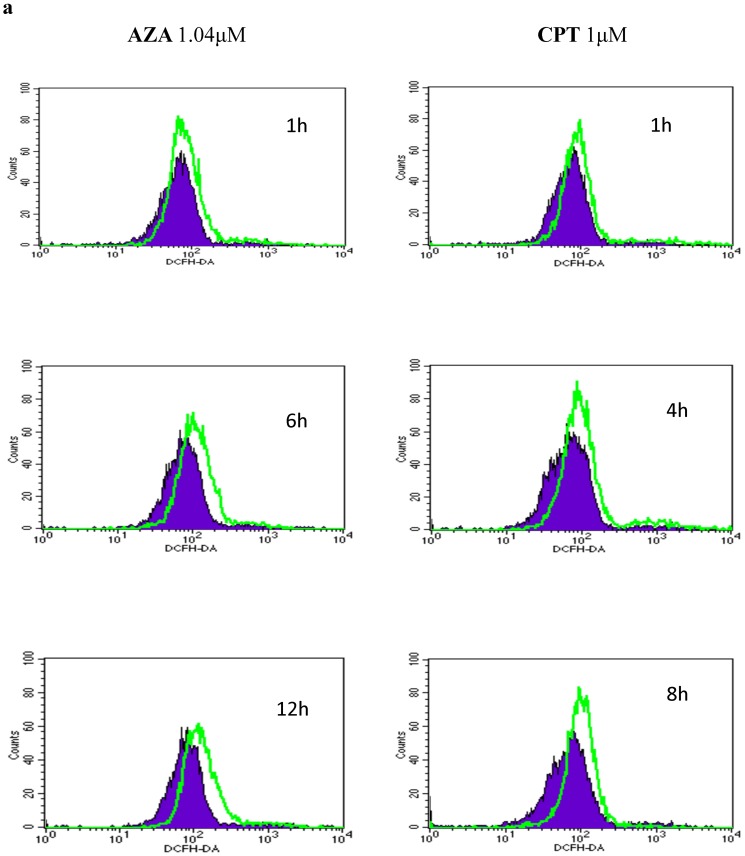
AZA and CPT induced increase in intracellular ROS. Cells treated with azadirachtin and camptothecin were stained with DCFDA and analyzed by flow cytometry. (a). Mitochondrial release of cytochrome c release after treated with azadirachtin and camptothecin at 0 h, 12 h and 24 h (b).

The export of cytochrome c from mitochondria during apoptosis proceeds in a two-step process: cytochrome-c first detaches from binding to cardiolipin and is subsequently released into the cytosol [42], [43]. The detachment of cytochrome-c from cardiolipin is triggered by an increase in intracellular ROS, which is one of the early events of apoptosis induced by a vast variety of agents [44], [45]. Therefore, the changes in ROS levels following azadirachtin and camptothecin treatment at different time intervals was analysed by DCFDA. As shown in Fig. 2b, flow cytometric analysis of DCFDA fluorescence revealed low ROS levels in the untreated cells with MFIs between 80.83 and 97.22. A profound increase (by 31.17% and 48.78%) in the levels of intracellular ROS was observed as early as 1 h into the azadirachtin and camptothecin treatments (AZA: MFI 106.02 and CPT: MFI 128.58). For azadirachtin treatment, cells with a higher MFI (144.54) further increased up to 24 h. However, the ROS levels in cells treated with camptothecin reached maximal levels at 4 h of treatment, which is 1.84 times higher than that observed in the control (MFI 164.87). There was an evident decrease in ROS levels 4 h later (MFI 124.01). Despite the ROS decrease, the ROS level after camptothecin treatment was higher than the control at all treated time points.

To define the role of mitochondria in apoptosis of Sf9 cells induced by both of these botanical chemicals, the mitochondrial release of cytochrome c was first analysed using western blotting. As shown in Fig. 2b, in treated Sf9 cells, cytochrome c was observed to be released from the mitochondria to the cytosol at 12 h after both treatments, which increased in a time-dependent manner in the cytosolic fractions. In contrast, in the mitochondrial fractions, cytochrome c levels began to decrease at 12 h post treatment, indicating that cytochrome c release from mitochondria into the cytosol started in an early phase during apoptosis and continued with apoptotic progress.

### 3.3 Activation of mitochondrial permeability transition pore (MPTPs) and the loss of mitochondrial membrane potential (MMP)

**Figure 3:**
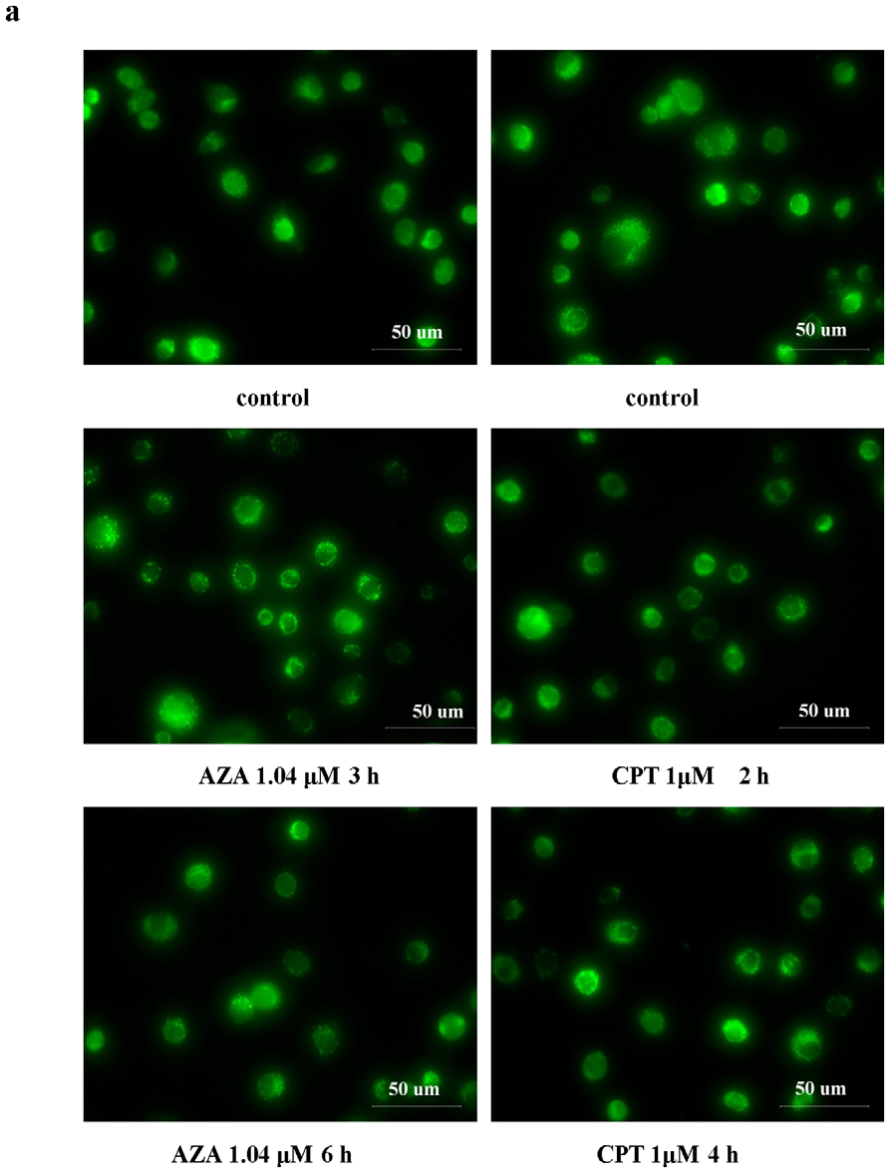
MPTP activation and MPT loss induced by AZA and CPT. (a). MPTP open induced by AZA 1.04 µM and CPT 1 µM at different times. The cells were loaded with calcein AM (cAM) and CoCl_2_ (cytosolic calcein quencher) to determine the calcein fluorescence in the mitochondria MMP. Bar =  50 µm (b). MMP loss of Sf9 cells after treated with AZA and CPT. Sf9 cells were stained with RH123, and observed under a fluorescent microscope Bar =  50 µm.

There are at least two proposed strategies to explain the regulation of cytochrome c release from mitochondria; one involves opening the permeability transition pore (PTP), mediated by mitochondrial Ca^2+^ overload and subsequent MMP loss [44], and the other is independent of PTP opening. To examine whether cytochrome c release in azadirachtin- and camptothecin-treated Sf9 cells involves MPTP opening, the MPTP activity was analysed by the calcein AM-CoCl_2_ method [46]. Prolonged MPTP opening results in dissipation of the MMP, so it can be used as a marker of MPTP activity. As shown in Fig. 3a, after treatment with azadirachtin and camptothecin for different amounts of time, the fluorescence of calcein in the cytoplasm had declined. The opening of MPTPs causes an efflux of calcein from the mitochondria to the cytoplasm, and the calcein was then quenched by CoCl_2_ in the cytoplasm.

As shown in Fig. 3b, the MMP (Δψm) of Sf9 cells after treatment with azadirachtin and camptothecin for different times was investigated using fluorescence microscopy. The control cells showed strong green fluorescence, which suggests intact MMP. The fluorescence intensity decreased when cells were treated with azadirachtin and camptothecin for 9 h and 4 h, which indicated a loss of MMP and mitochondrial depolarisation.

### 3.4 Induction of mitochondria swelling and decline in mitochondria respiration

Swelling of the mitochondrial inner membrane can damage the outer membrane without breaching the integrity of the inner membrane, which is one essential way for cytotoxic protein release [47]. Mitochondria are dynamic structures capable of high rates of metabolism. Thus the rate at which metabolites can cross the outer membrane will limit mitochondria metabolic rates which will also greatly influence metabolic rates in the rest of the cell because of the highly intertwined nature of the two metabolic systems [48]. Mitochondrial respiration control ratio (RCR) measurements can determine the intactness of the outer membrane [49].

**Figure 4:**
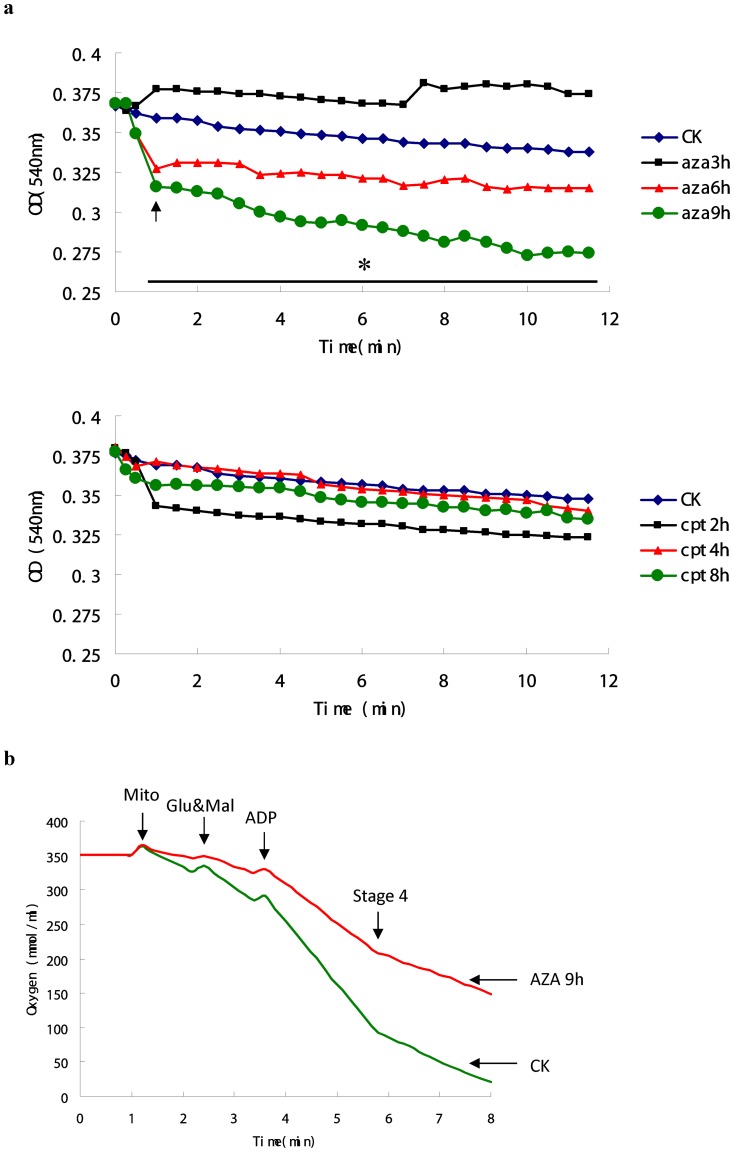
Effect of azadirachtin and camptothecin on mitochondrial function. (a) AZA- and CPT-induced swelling in isolated mitochondria. AZA- and CPT-induced swelling was measured under standard incubation conditions as described under section 2.8. AZA (1.04 µM) or CPT (1 µM) were included as indicated. After obtaining the baseline for ∼1 min, mitochondrial swelling was monitoring absorbance decline at 540 nm (n = 4; * = p<0.05). (b) AZA- and CPT -induced damage to mitochondrial respiratory function. Oxygen consumption rate of the isolated mitochondria was measured in the presence of glutamate (5 µM) and malate (5 µM) as respiratory substrates.

As shown in Fig. 4a, after treatment of 6 h with 1.04 µM AZA, the OD value of Sf9 mitochondria decline to a certain extent, and show significant decline after treatment of 9 h. By contrast, isolated mitochondria show a slight decline in absorbance at 540 nm only after 2 h treatments of CPT-induced group. It is suggested that AZA induced mitochondrial swelling of Sf9 cell line. Furthermore, AZA inhibited the oxygen consumption rate of Sf9 cells significantly (Fig. 4b), which also indicated the same inhibition effect on mitochondrial respiration (Table 1). However, it is not the case in CPT-treated group (Fig. 4b, Table 1).

**Table 1:** The effect of AZA and CPT on mitochondrial respiration function.

	Stage 3	Stage 4	RCR
CK	90.29±0.39	32.98±0.87	2.74±0.064
AZA	56.00±0.36 *	26.44±0.72	2.12±0.057 *
CPT	80.46±0.26	30.55±0.47	2.64±0.041

Note: Respiratory control ratio (RCR) is the ratio of State 3 (ADP stimulated) respiration to State 4 (resting) respiration. (n = 4; * = p<0.05).

### 3.5 The role of MPTPs in Sf9 apoptosis

#### 3.5.1 The effect of CsA on cell viability of Sf9 cells

To investigate the role of MPTPs in Sf9 apoptosis induced by azadirachtin and camptothecin, CsA, which block PTP by binding with cyclophilin D, a known participant in the PTP formation [50] was used to treat Sf9 cells prior to treatments. Maintenance of calcein fluorescence indicates less active MPTP opening, as more calcein is being trapped in the mitochondria. Inhibition of MPTP opening by CsA may allow mitochondria to resist depolarisation and prevent proapoptotic molecules from exiting the mitochondria.

**Figure 5:**
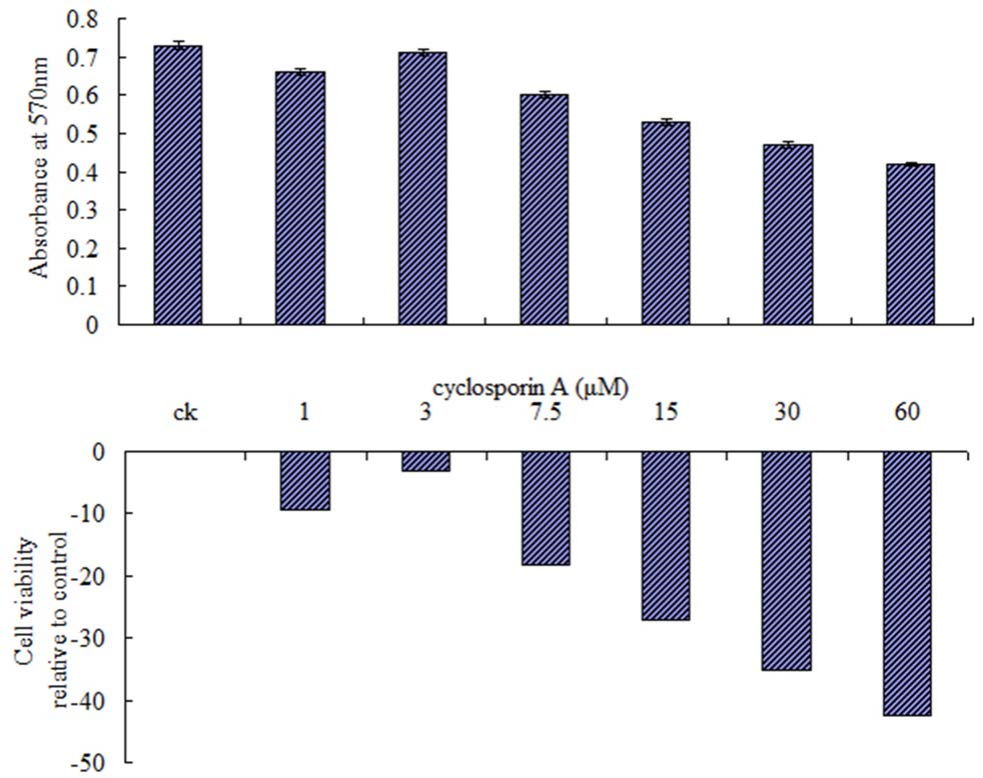
Effect of CsA at different concentrations on cell viability of Sf9 cells. 0.01×10^6^ cells were pre-cultured for 24 h in and subsequently exposed with various doses of CsA for 1 h using 0.5% DMSO as a control. Percentage survival was determined as per the formula (Absorbance of drug-treated cells/Absorbance of control cells) ×100 and compared with control, untreated cells regarded as 100%. Data of absorbance shown are the mean±S.E.

The immunosuppressive activity of CsA is exerted through the complex formed between CsA and cyclophilin A (CypA) [51]. However, as an effective immunosuppressive agent, CsA can induce apoptosis at a higher concentration. It is important to define an appropriate concentration for different cells when treated for the first time. The MTT assay was used to determine the appropriate concentration of CsA, which is able to suppress MPTP opening efficiently with less inhibition of cell proliferation. Fig. 5 shows the viability of Sf9 cells after treatment with different concentrations of CsA for 1 h. The results showed that treatment with 3 µM CsA was less inhibitory than treated with other concentrations (absorbance at 570 nm = 0.71, cell viability = 96.95%). Thus, 3 µM CsA was used for all following MPTP activation experiments.

#### 3.5.2 MPTP opening and mitochondrial membrane potential (Δψm) loss

**Figure 6:**
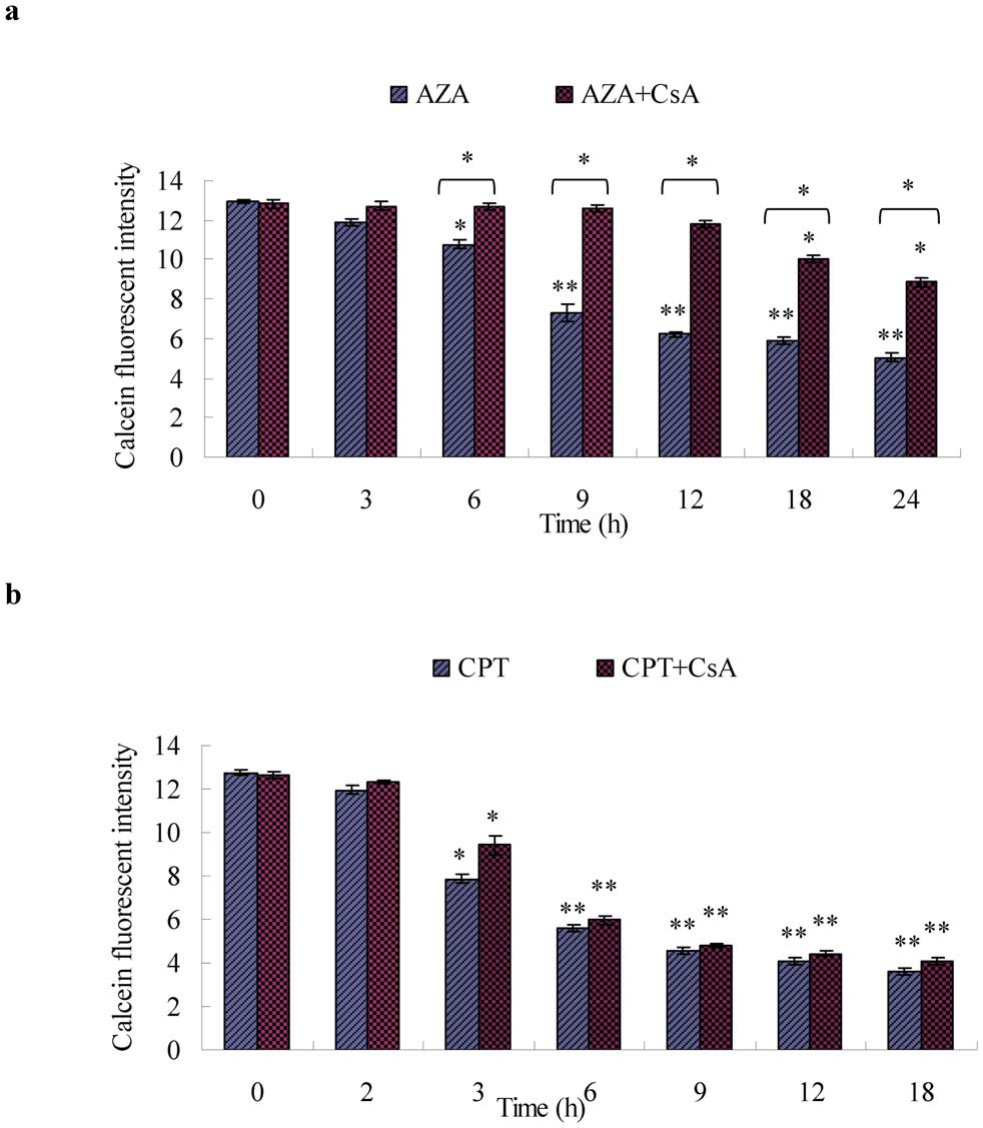
MPTP activation induced by AZA+ CsA and CPT+ CsA. (a) MPTP open induced by AZA and AZA+ CsA -treatments at 3 h, 6 h, 9 h, 12 h, 18 h, 24 h, respectively. (b) MPTP open induced by CPT and CPT+ CsA -treatments at 2 h, 3 h, 6 h, 9 h, 12 h, 18 h, respectively. The MPTP was monitored by quantifying the fluorescence of calcein in the mitochondria of Sf9 cells by flow cytometry. The cells were then loaded with calcein AM (cAM) and CoCl_2_ (cytosolic calcein quencher) to determine the calcein fluorescence in the mitochondria MMP. Results represent means ±SE (n = 4). Different asterisks above the SE bars represent statistically significant difference (*p<0.05 **p<0.01) compared with control when the data were analyzed by ANOVA followed by Duncan's multiple range test (DMRT).

To further investigate the role of MPTPs in the mitochondrial/caspase apoptotic pathway, the effects of CsA, a specific MPT inhibitor of MPTP opening and Δψm loss, were detected by flow cytometry. Fig. 6 shows the effect of CsA on the MPTP opening induced by azadirachtin and camptothecin in Sf9 cells. Compared to the controls (AZA: MFI 12.94 and CPT: MFI 12.76), both treatments showed significantly decreased fluorescence intensity at 9 h and 3 h (AZA: MFI 7.31 and CPT: MFI 7.86), respectively. The calcein fluorescence decreased significantly after 12 h and 6 h or more, and the calcein MFIs decreased to 4.97 and 3.62 at 24 h and 18 h post treatment, respectively. The results also showed that CsA could suppress the azadirachtin-induced opening of MPTPs after 12 h (Fig. 6a). The calcein MIFs did not significantly decrease until after 18 h or more. However, as shown in Fig. 6b, CsA could not inhibit the camptothecin-induced opening of MPTPs. Similar to the group of cells treated with camptothecin alone, the calcein MIFs only decreased significantly after 3 h or more.

**Figure 7:**
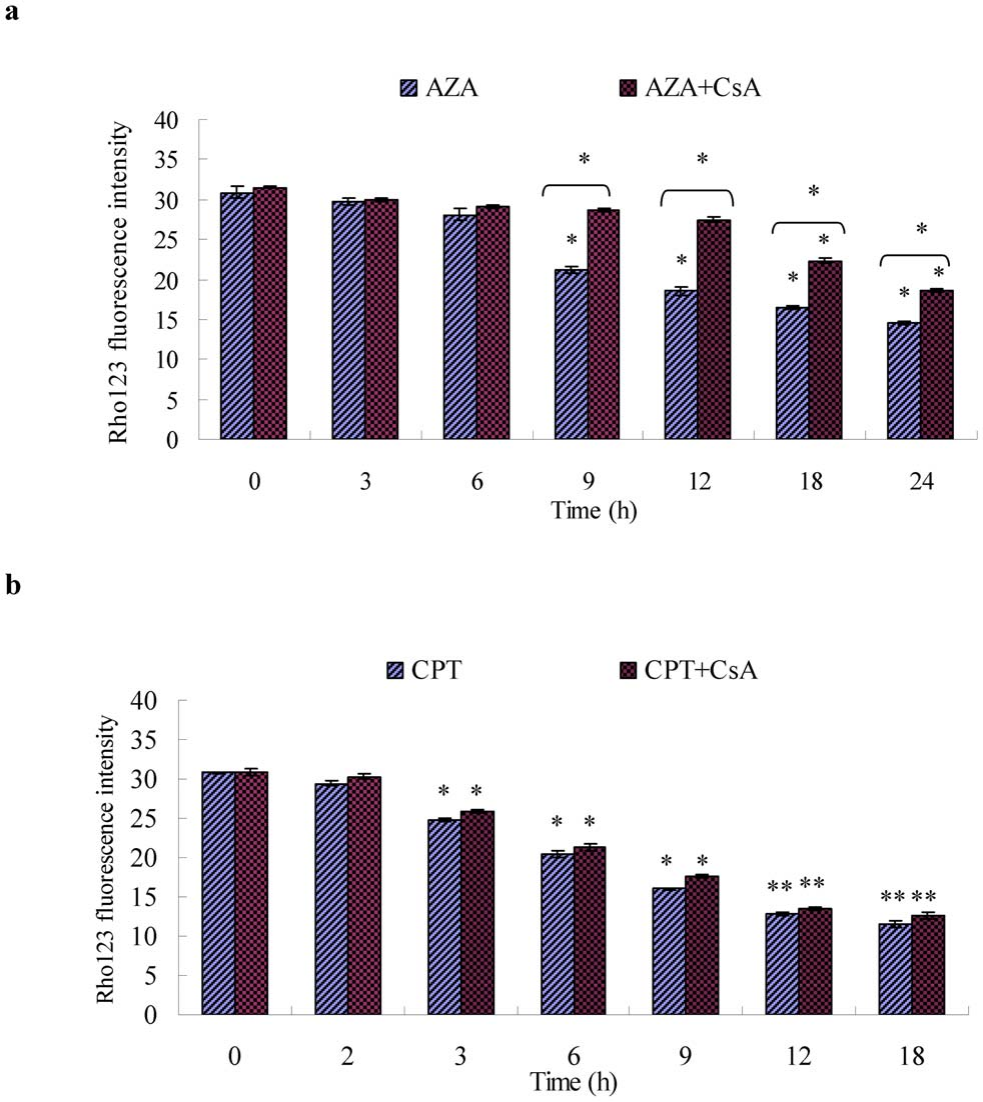
Effect of CsA on ΔΨm loss of Sf9 cells treated with AZA and CPT. (a) ΔΨm loss induced by AZA and AZA+ CsA -treatments at 3 h, 6 h, 9 h, 12 h, 18 h and 24 h, respectively. (b) ΔΨm loss induced by CPT and CPT+ CsA-treatments at 2 h, 3 h, 6 h, 9 h, 12 h and 18 h, respectively. Results represent means ±S.E (n = 4). Different asterisks above the S.E bars represent statistically significant difference (*p<0.05, **p<0.01), when the data were analyzed by ANOVA followed by DMRT.

Mitochondria isolated from a variety of sources can show a sudden increase in the permeability of the inner mitochondrial membrane to solutes with a molecular mass of less than 1,500 Da, which results in the loss of Δψm, mitochondrial swelling, and rupture of the outer mitochondrial membrane [52], [53]. Therefore, the role of the MPT in apoptosis induced by azadirachtin and camptothecin was subsequently assessed by evaluating the effect of CsA on changes in Δψm. As shown in Fig. 7a, compared to the control (MFIs = 30.89), the Δψm significantly decreased at 9 h (MFI = 21.22) after treatment with azadirachtin alone. However, when pre-treated with CsA, the Δψm of Sf9 cells did not significantly decrease until 18 h after azadirachtin treatment. There are significant differences between the Δψm of groups treated with CsA and azadirachtin and those treated with azadirachtin alone after 9 h or more. Fig. 7b showed that Δψm significantly decreased after 3 h or more, when treated with camptothecin alone. However, unlike the groups treated with CsA and azadirachtin, CsA alone could not delay the onset of camptothecin-induced MMP loss, and complete inhibition of MMP dissipation was not observed (Fig. 7b). Treatments with CsA and camptothecin also significantly decreased the Δψm after 3 h or more. CsA did not influence MPTP opening or camptothecin-induced MMP loss.

#### 3.5.3 Cytochrome c release and caspase activation

**Figure 8:**
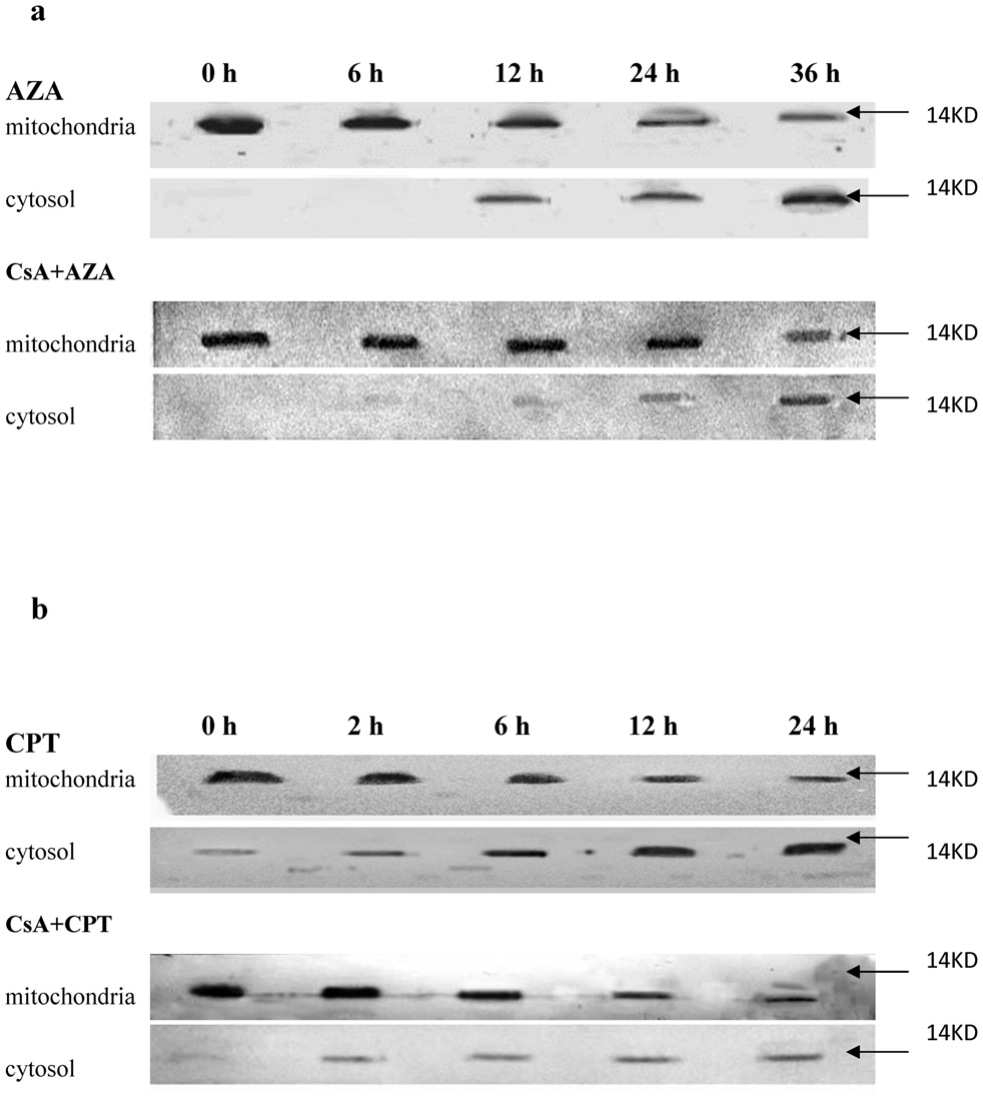
Cytochrome c release from mitochondria to cytosol after treated with AZA and CsA+ AZA (a), CPT and CsA+ CPT (b) at various time. The translocation of cytochrome c was detected by western blotting.

An increase in the permeability of the outer mitochondrial membrane is crucial for apoptosis to occur, resulting in the release of several apoptogenic factors, such as cytochrome c, into the cytoplasm [54]. In the present study, the effect of CsA on cytochrome c released was also analysed by western blotting, which is more accurate than the method described in section 3.2. As shown in Fig. 8a, CsA efficiently suppresses azadirachtin-induced cytochrome c release. Cytochrome c release in Sf9 cells treated with CsA and azadirachtin was not observed until 24 h post treatment, which is 12 h later than in the cells treated with azadirachtin alone (Fig. 8a). This suggests that the azadirachtin-induced cytochrome c release in Sf9 cells is dependent on MPTPs to some degree. However, as shown in Fig. 8b, mitochondrial cytochrome c release in Sf9 cells can be observed as early as 2 h after treatment with CsA and camptothecin, which is only slightly suppressed by CsA and almost the same as the Sf9 cells treated with camptothecin alone. Furthermore, cytochrome c releases induced by camptothecin and CsA plus camptothecin were both earlier than the mitochondrial membrane potential loss, which did not significantly decrease until 3 h after treatment (Fig. 8b). This result suggests that the camptothecin-induced cytochrome c release was independent of MPTPs and insensitive to CsA. In addition to CsA-sensitive and Ca^2+^-dependent MPT, the existence of a CsA-insensitive MPT has also been suggested [55], [56].

**Figure 9:**
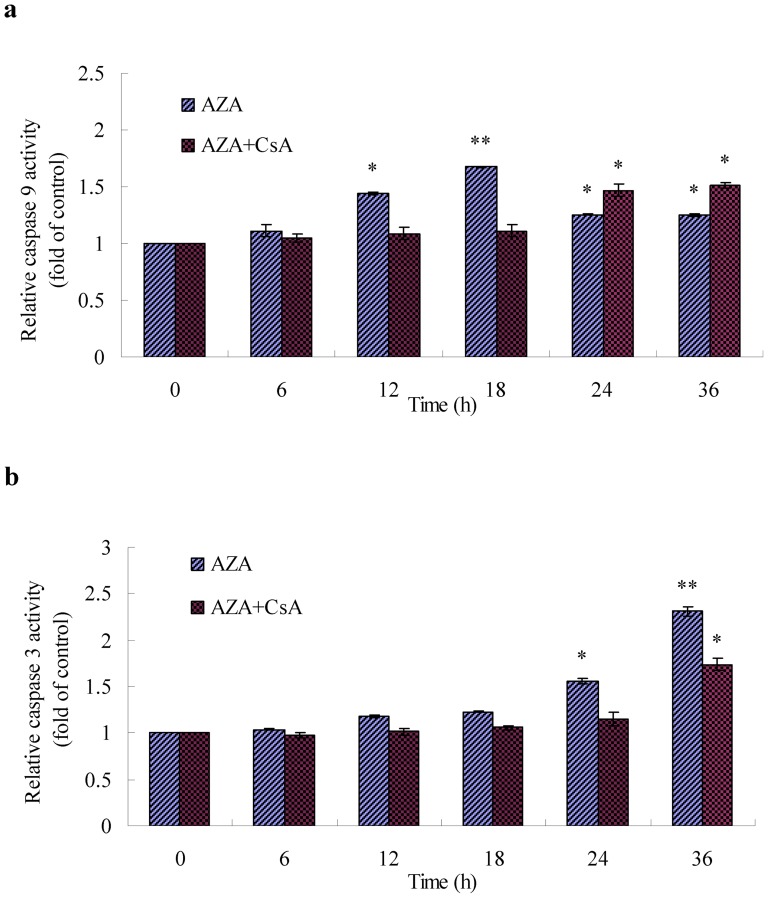
Effects of AZA and CPT on enzyme activities of caspase-9 and caspase-3 in Sf9 cells. a, b, c, and d showed the results of treatments with AZA, AZA+ CsA, CPT and CPT + CsA,, respectively. Results represent means ±S.E (n = 4). Different asterisks above the S.E bars represent statistically significant difference (*p<0.05, **p<0.01), when the data were analyzed by ANOVA followed by DMRT.

To further define the role of cytochrome c in azadirachtin- and camptothecin- induced apoptosis, caspase activation was studied in cells treated with azadirachtin and camptothecin in the presence/absence of inhibitors of CsA. As shown in Fig. 9a and Fig. 9b, CsA can suppress the activation of caspase-9 and caspase-3 in azadirachtin-treated groups. When treated with azadirachtin alone, caspase-9 and caspase-3 activities increased with time and reached maximal levels at 18 h (1.67 fold over control) and 36 h (2.31 fold over control), respectively. When treated with CsA plus azadirachtin, caspase-9 and caspase-3 activities were significantly suppressed; both reached maximal levels at 36 h and decreased to 1.51 fold and 1.74 fold over the control, respectively (Fig. 9a, b). As shown in Fig. 9c and Fig. 9d, the activities of caspase-9 and caspase-3 in Sf9 cells treated with camptothecin were also suppressed by CsA to a small extent. When treated with camptothecin alone, caspase-9 and caspase-3 activities increased in a time-dependent manner, which maximised at 12 h (1.76 fold over control) and 24 h (2.75 fold over control) after treatment (Fig. 9c, d). However, the activities of caspase-3 and caspase-9 significantly increased at 18 h (1.73 fold over control) and 24 h (2.08 fold over control) of treatments when treated with CsA and camptothecin (Fig. 9c, d). Despite suppression by CsA, the activities of caspase-9 and caspase-3 in the camptothecin-treated cells showed the same increase. The activities of caspase-9 significantly increased as early as 2 h after treatment, either in the presence or absence of CsA. And the corresponding significant increase of caspase-3 activities were both showed as early as 12 h after the same treatment.

#### 3.5.4 Effect of CsA on apoptosis induction of Sf9 cells

**Figure 10:**
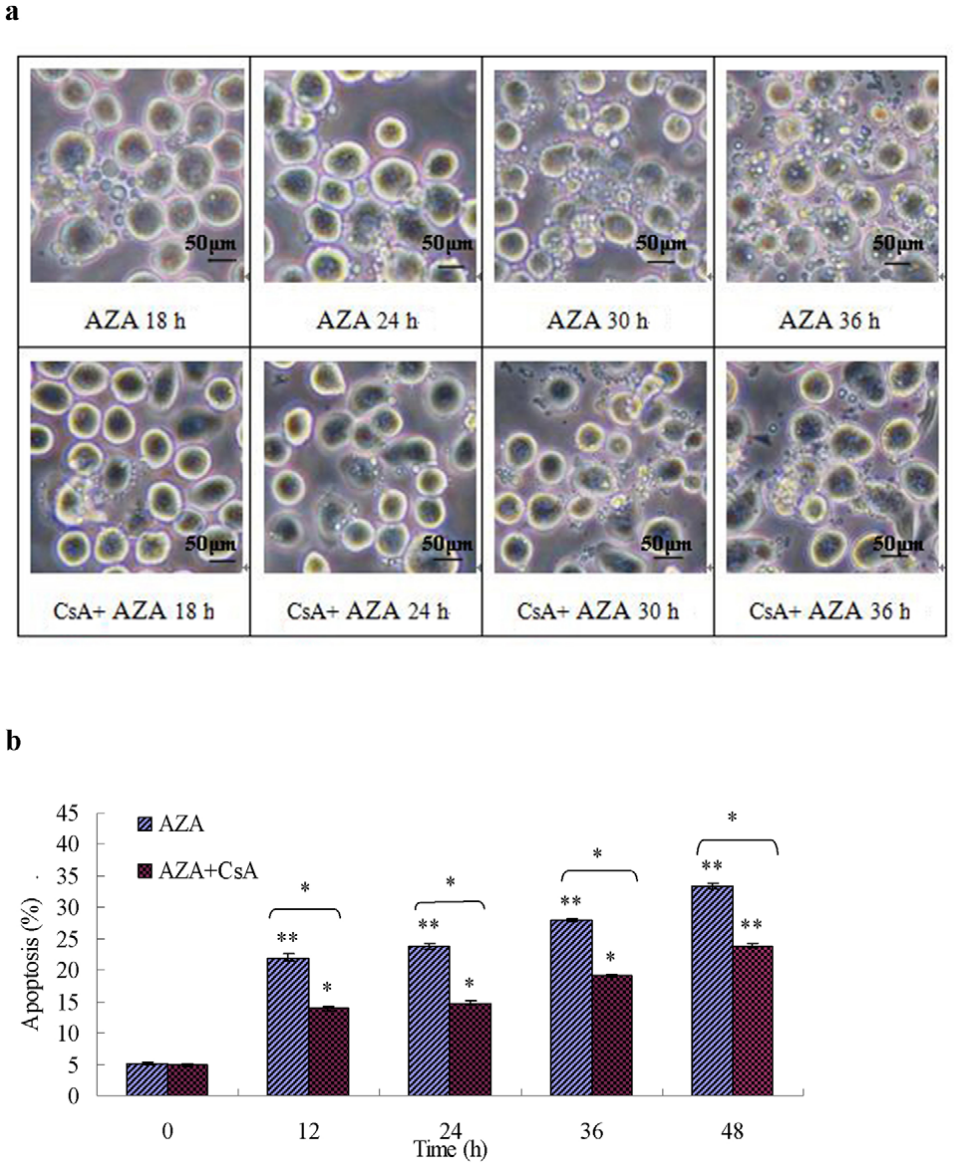
Effect of CsA on apoptosis induction of Sf9 cells. (a) and (b) showed the morphological characteristics with apoptotic body formation and apoptotic percentage of Sf9 cells after treated with AZA and AZA+ CsA, respectively. (c) and (d) showed the morphological characteristics with apoptotic body formation and apoptotic percentage of Sf9 cells after treated with with CPT and CPT+ CsA. Results represent means ±S.E (n = 4). Different asterisks above the S.E bars represent statistically significant difference (*p<0.05, **p<0.01), when the data were analyzed by ANOVA followed by DMRT. Bar (a), 50 µm; Bar (b), 50 µm.

Fig. 10 shows the effect of CsA treatment on azadirachtin- and camptothecin-induced apoptosis in Sf9 cells using IPCM and flow cytometry, respectively. Morphological study from IPCM showed that pre-treatment with CsA can suppress the apoptosis of Sf9 cells induced by azadirachtin. However, this is not the case following camptothecin treatments. CsA can recover the morphological damage and inhibit the formation of an apoptosis body in Sf9 cells that were induced by azadirachtin (Fig. 10a). Furthermore, the flow cytometric analysis also showed that CsA can significantly suppress apoptosis in azadirachtin-treated Sf9 cells (Fig. 10b). However, the suppressive effect of CsA on apoptosis in camptothecin-treated Sf9 cells is less evident, as the number of apoptotic cells decreases slightly but not significantly (Fig. 10c). The flow cytometric analysis also found that after 36 h of treatment, the percentage of apoptotic cells in camptothecin-treated and CsA and camptothecin-treated groups are almost the same (Fig. 10d).

## Discussion

Our earlier work has first defined the apoptosis-inducing effect of azadirachtin on Lepidopteran SL-1 cells [16]–[17]. The present study aims to further investigate the mechanism of azadirachtin-induced apoptosis in Lepidopteran cell lines and the role of MPTPs in apoptosis of insect cell line. Camptothecin was used as a positive apoptosis inducer throughout the experiment [22]. The present study showed that the release of cytochrome c into the cytosol is an important event during Lepidopteran apoptosis induced by the two botanical chemicals, which is similar to stress-induced apoptosis in mammalian cells. Followed by the cytochrome c release, the activation of initiator and effector caspases (caspase-9 and -3) was also detected. However, most importantly, in azadirachtin-induced apoptosis, the cytochrome c release accompanied with ROS generation, mitochondrial membrane potential loss, and MPTP opening could not be entirely inhibited but strongly delayed by the MPTP inhibitor CsA. Furthermore, we found that the mitochondria/caspase apoptotic pathway of Sf9 cells induced by camptothecin is insensitive to CsA.

It is well known that mammalian cells possess two major apoptotic signalling pathways, known as the intrinsic pathway and the extrinsic pathway [57]. Mitochondria are central to the intrinsic apoptosis pathway, which determines whether cells survive or die [58], [59]. Recent studies have confirmed the intrinsic pathway involves an increase in outer mitochondrial membrane permeability that leads to the release of various proteins from the intermembrane space into the cytoplasm, including apoptogenic molecules such as cytochrome c, Smac/Diablo, HtrA2 (Omi), AIF, and DNase G [60]–[61]. Cytochrome c is considered to be one of the most important proteins in the mitochondrion due to its important role in apoptosis [59].

Cytochrome c is associated with cardiolipin, a signature mitochondrial lipid, which is enriched in the inner membrane and in the contact points between the inner and outer membranes [62]. Cytochrome c must first dissociate from cardiolipin to completely release from the mitochondrion. Dissociation of cytochrome c from cardiolipin is primarily achieved by the breach of the electrostatic/hydrophobic interactions caused by cardiolipin peroxidation by reactive oxygen species (ROS) [42], [63]. In Sf9 cells treated with azadirachtin and camptothecin, significant increases in intracellular ROS were observed as early as 1 h post treatment (Fig. 2a), and the release of mitochondrial cytochrome c into the cytosol was subsequently detected by 12 h post treatment (Fig. 2b). These observations strongly implicate ROS induction as a preceding event leading to cytochrome c release and suggest the involvement of mitochondria in Sf9 cell apoptosis induced by azadirachtin and camptothecin.

Whether cytochrome c is released from the mitochondria in insect apoptosis has also been a source of controversy. In *Drosophila* system, it is supposed that there are both cytochrome c-dependent and -independent mechanisms for apoptosis regulation and the models of regulation may be cell-type dependent. Some cell types, such as *Drosophila* retinal cells or differentiating sperm cells require cytochrome c [64]. But in most *Drosophila* cells, cytochrome c is not required for apoptosis and caspase activation [65]–[68]. The role of cytochrome c during *Drosophila* cell apoptosis should be investigated in more cell lines as well as tissue types. Unlike *Drosophila*, apoptosis induced with different stimuli, such as UV, viral infection or RNA/DNA syntheses inhibitors always involved cytochrome c release in different Lepidopteran species. Apoptosome formation and caspase activation in Lepidopteran cell extracts was found to be exclusively dependent on cytochrome c release [13], [69], [70]. It seems the intrinsic apoptosis pathway of Lepidopteran cells in is similar to mammalian cells. Compared to *Drosophila* and humans, the apoptosis regulation mechanism of Lepidopteran cells is much further away from demonstrated, because only a few genes in apoptosis regulation pathway have been isolated in Lepidopteran cells. More genes such as the initiator caspases and Bcl-2 familiy need to be identified. And the characterization of apoptosome formation as well as mechanisms of caspase activation also needs further study [68].

Corroborating these recent reports in Lepidopteran cell lines, our study also confirms that cytochrome c release (Fig. 2b, Fig. 8) into the cytosol is an earlier and important event during Lepidopteran insect cell apoptosis induced by azadirachtin and camptothecin. The results of azadirachtin treatment indicated that the inhibition of cytochrome c release by CsA directly results in the suppression of the activities of initiator and effector caspases (Fig. 8a, b). An indirect interpretation is that the release of cytochrome c induced by camptothecin is insensitive to CsA (Fig. 8c, d). In addition, the subsequent activation of caspases and the morphological and flow cytometry analysis of apoptosis (Fig. 10) also further demonstrated the role of cytochrome c release in the apoptosis of Sf9 cells induced by camptothecin.

To further determine the intactness of mitochondria outer membrane, the mitochondrial swelling and mitochondrial respiratory activity were also detected in the present paper. Mitochondrial membrane swelling (MMS) is often measured as an indication of PTP opening [71]. Our results found that both AZA and CPT can induce mitochondrial swelling in Sf9 cell line to a different degree (Fig. 4a). However, CPT showed no significant inhibition effect on mitochondrial respiration control ratio, which was inhibited significantly by AZA. Consist with our founding, Bredholt et al (2009) [72] also found that CPT showed no inhibition effect on the oxygen consumption of MOLM-13 and MV-4-11 cells. In addition, Sharov et al (2007) [73] found that CsA can improve mitochondrial respiratory function to attenuate mitochondrial permeability transition in cardiomyocytes isolated from dogs with heart failure, which also implies the inhibition effect of CsA on AZA-induced Sf9 cells apoptosis.

Recent studies also found that after detaching from the oxidised cardiolipin, cytochrome c releases from mitochondria through two distinct processes, which are either MPTP-dependent or -independent. In the MPTP-dependent cytochrome c release, cytochrome c releases from mitochondria following MPTP opening and could be detected using the specific probe calcein-AM [44], [45]. In the present study, both camptothecin and azadirachtin could induce the opening of MPTPs, which is closely followed by the loss of mitochondrial membrane potential during the initial few hours of treatment (Fig. 3). However, MMP induced by camptothecin is insensitive to CsA, a specific MPTP inhibitor. Furthermore, cytochrome c release induced by camptothecin occurs earlier than the MPTP opening, regardless of the presence or absence of CsA. MMP leading to the loss of membrane potential (Δψm) was not observed before 3 h of camptothecin treatment (Fig. 3b, 6b, and 7b), whereas cytochrome c release was evident as early as 2 h post treatment (Fig. 8 c, d). Similar to our present study, Sen et al. [74] also found that the loss of mitochondrial membrane potential and cytochrome c release in apoptosis of *Leishmania donovani* induced by camptothecin are insensitive to CsA. However, in mammalian cells, CsA can significantly reduce mitochondrial depolarisation (Δψm loss) in CPT-induced apoptosis [75].

By contrast, the cytochrome-c release induced by azadirachtin showed a perfect cause-effect relationship with the MMP. Suppression of MMP by CsA directly results in the inhibition of cytochrome c release (Fig. 8a, b). Although not entirely inhibited by CsA, MPTP opening and Δψm were strongly suppressed and delayed for more than 9 h (Fig. 3a, 6a, and 7a). Consequently, cytochrome c release was observed 12 h later, which also resulted in the suppression of caspase activity and recovery of apoptosis induction (Fig. 9a, c, and 10a, c).

In regards to the different effects of CsA on the mitochondrial signal transduction pathways induced by the two botanical chemicals, our previous experiments detected the effects of azadirachtin and camptothecin on the activity of Topo I (data not shown). No inhibition was observed, even when the cells were treated with 90 µg/mL azadirachtin, while obvious inhibition was observed when treated with 0.35, 3.5, and 21 µg/mL of camptothecin.

Our study provides important insight into the mitochondria-mediated events during Lepidopteran cell apoptosis induced by two botanical chemicals. It is clear from this study that both MPTP dependent- and independent-cytochrome c release mechanisms exist in Lepidopteran cells. Furthermore, cytochrome c release into the cytosol is thought to be an essential and possibly exclusive event for the activation of caspases. In summary, the present study demonstrates significant similarities between stress-induced apoptosis in Lepidopteran Sf9 cells and mammalian cell apoptosis, while showing distinctive differences from earlier reports in *Drosophila*. Further studies for the characterisation of apoptosome formation and regulation of initiator caspases would further aid our current understanding of the process of cell death in these insect cells.
