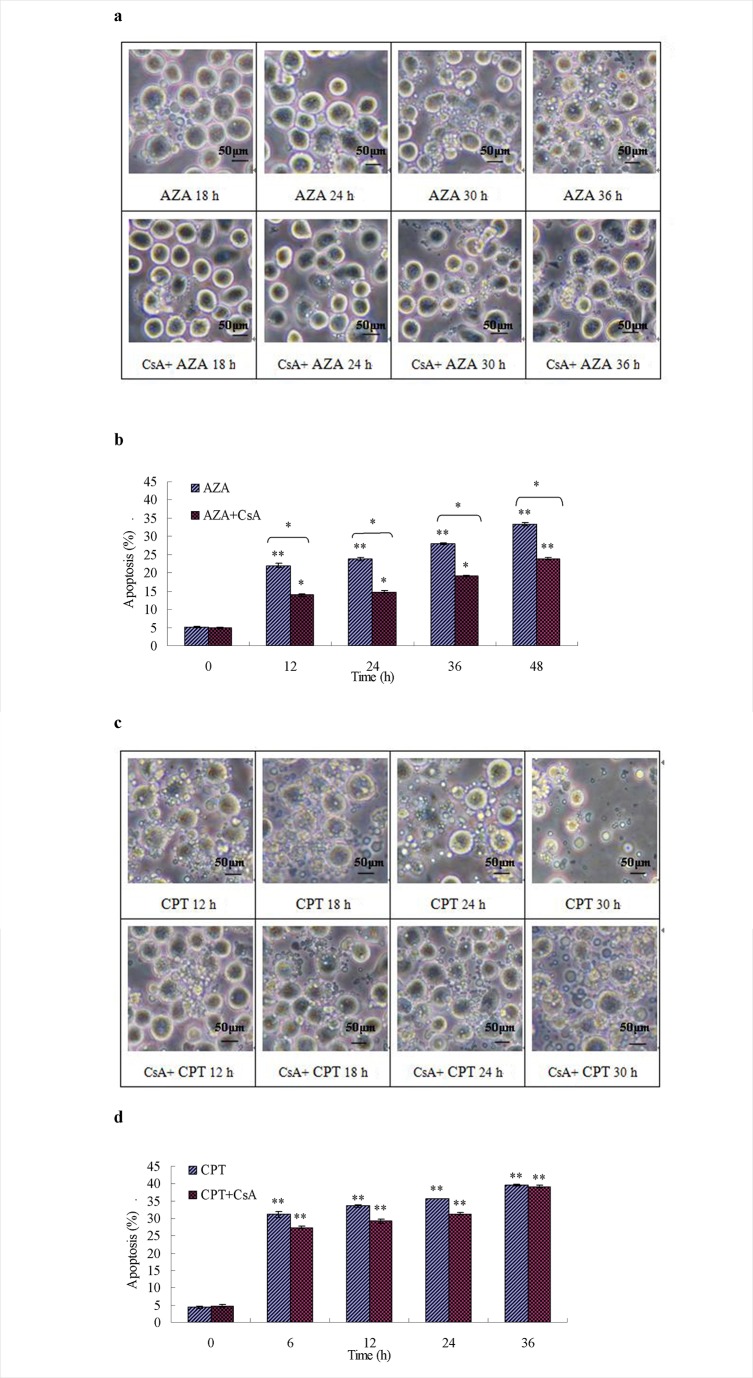# Correction: The Mitochondria-Mediate Apoptosis of Lepidopteran Cells Induced by Azadirachtin

**DOI:** 10.1371/annotation/c51cc574-68e6-4389-b4d3-df8b6227035d

**Published:** 2013-10-24

**Authors:** Jingfei Huang, Chaojun Lv, Meiying Hu, Guohua Zhong

Incomplete figures were published for Figures 2, 3, 9 and 10. The complete figures can be found here:

Figure 2: 

**Figure:**
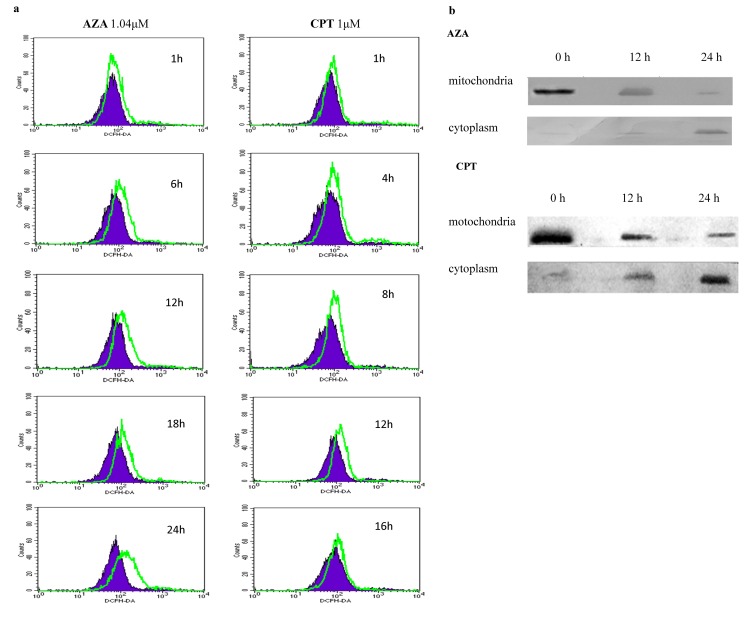


Figure 3: 

**Figure:**
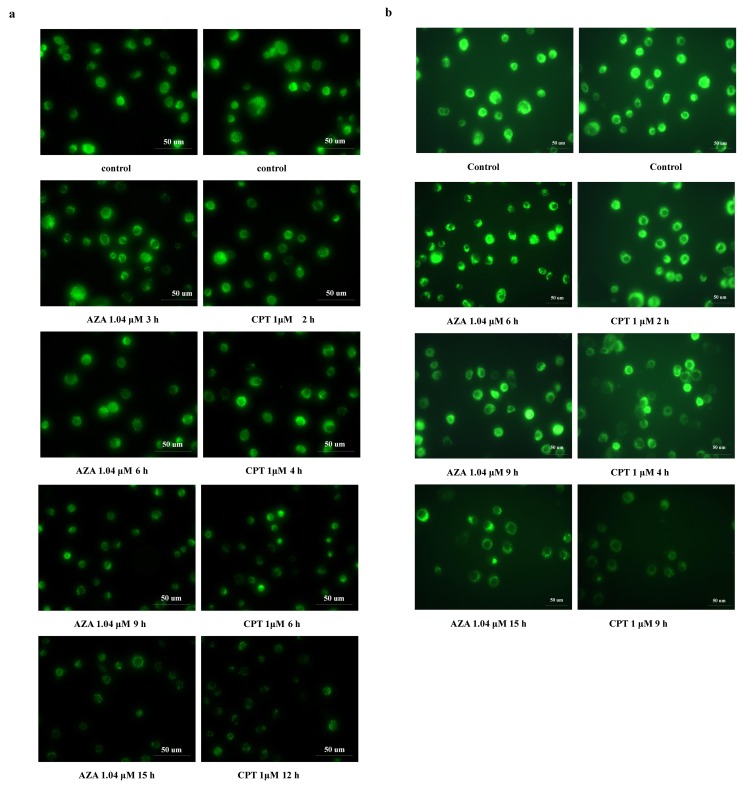


Figure 9: 

**Figure:**
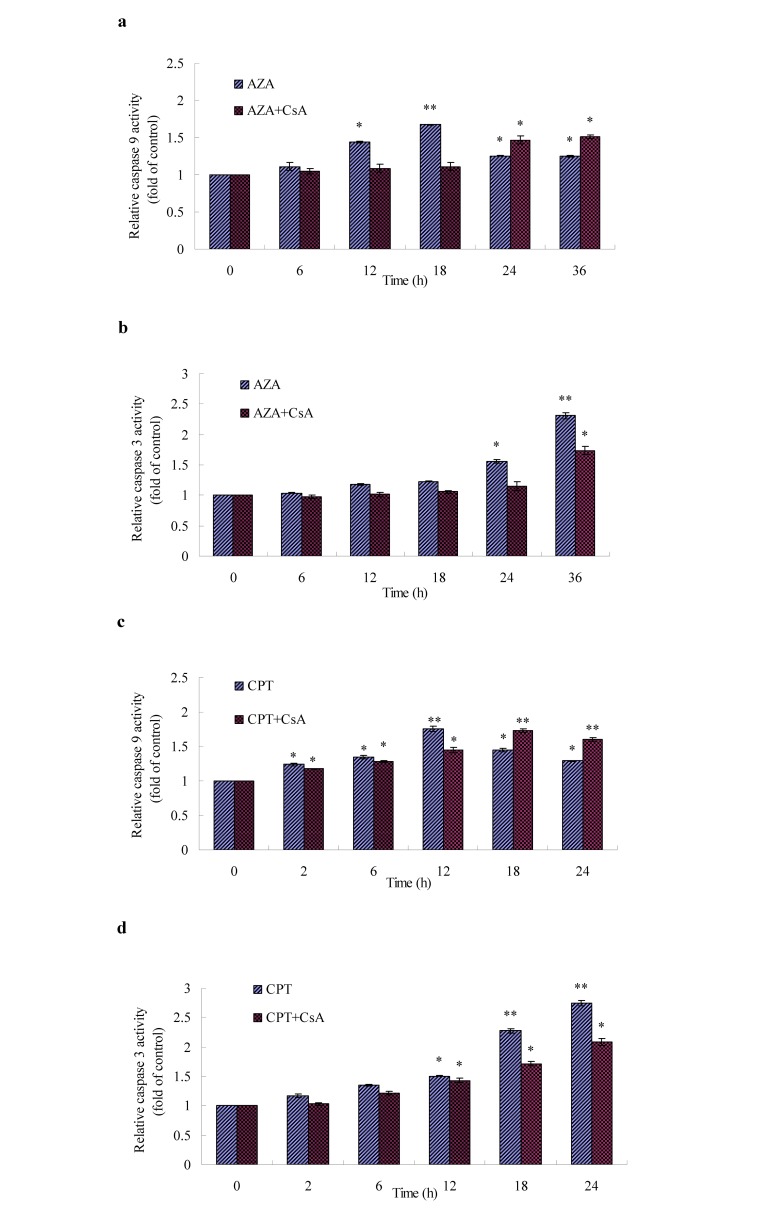


Figure 10: 

**Figure:**